# Microbial Selection and Survival in Subseafloor Sediment

**DOI:** 10.3389/fmicb.2019.00956

**Published:** 2019-05-14

**Authors:** John B. Kirkpatrick, Emily A. Walsh, Steven D’Hondt

**Affiliations:** ^1^Graduate School of Oceanography, The University of Rhode Island, Narragansett, RI, United States; ^2^The Evergreen State College, Olympia, WA, United States

**Keywords:** marine sediment bacteria, marine sediment archaea, deep biosphere, microbial selection, 16S rDNA, U1343, NGHP-14

## Abstract

Many studies have examined relationships of microorganisms to geochemical zones in subseafloor sediment. However, responses to selective pressure and patterns of community succession with sediment depth have rarely been examined. Here we use 16S rDNA sequencing to examine the succession of microbial communities at sites in the Indian Ocean and the Bering Sea. The sediment ranges in depth from 0.16 to 332 m below seafloor and in age from 660 to 1,300,000 years. The majority of subseafloor taxonomic diversity is present in the shallowest depth sampled. The best predictor of sequence presence or absence in the oldest sediment is relative abundance in the near-seafloor sediment. This relationship suggests that perseverance of specific taxa into deep, old sediment is primarily controlled by the taxonomic abundance that existed when the sediment was near the seafloor. The operational taxonomic units that dominate at depth comprise a subset of the local seafloor community at each site, rather than a grown-in group of geographically widespread subseafloor specialists. At both sites, most taxa classified as abundant decrease in relative frequency with increasing sediment depth and age. Comparison of community composition to cell counts at the Bering Sea site indicates that the rise of the few dominant taxa in the deep subseafloor community does not require net replication, but might simply result from lower mortality relative to competing taxa on the long timescale of community burial.

## Introduction

Marine sediment provides an ideal opportunity to test parameters that drive microbial selection in a relatively stable environment on very long timescales (1000s to millions of years) (Biddle et al., 2011a). Marine sedimentary microbes are globally abundant, with their total number roughly equal to the combined numbers of bacteria and archaea in the ocean (10^29^ cells) (Kallmeyer et al., 2012). They are important in nutrient cycling (Codispoti et al., 2001), greenhouse gas production, and carbon storage or release over geological time scales (Hedges and Keil, 1995). Despite their abundance and their biogeochemical significance, marine sedimentary microbes are subject to extremely low fluxes of energy per cell (D’Hondt et al., 2002, 2014; Price and Sowers, 2004; Røy et al., 2012).

Previous studies collectively provide broad evidence of taxonomic selection in subseafloor sediment. Bacterial diversity decreases with increasing sediment age (Walsh et al., 2015, 2016; Nunoura et al., 2016) and broadly defined taxonomic groups (e.g., *Chloroflexi*, *Deltaproteobacteria*, *Firmicutes*) have consistently been identified in subseafloor sediment of many different regions (e.g., Inagaki et al., 2006; Biddle et al., 2011b; Briggs et al., 2012; Mills et al., 2012; Bienhold et al., 2016; Nunoura et al., 2016; Walsh et al., 2016; Labonté et al., 2017). Microbial genes have been shown to be expressed deep in sediment columns, indicating differential microbial responses over time (Orsi et al., 2013). However, community responses to selective pressure, patterns of succession, and the extent of endism at finer taxonomic levels in subseafloor sediment remain largely unknown.

Changes in community composition with increasing sediment depth result from the combination of (i) subseafloor selection over geologic time and (ii) changes in initial seafloor community composition over time. Paleoceanographic studies routinely document the colonization age of subseafloor communities (when they were deposited on the seafloor) by determining the absolute age of marine sediment layers. This has recently been to extended microbial DNA studies, suggesting that near-seafloor events drive community variation that can persist over time (Harrison et al., 2018). Given initial colonization age and subseafloor sedimentary limits on diffusion and on energy for motility (Hoehler and Jørgensen, 2013), the consequences of microbial selection within sediment may be illustrated by changes in overall community composition and/or richness with increasing sediment age.

A very wide range of sediment ages (10^2^ to 10^6^ years) is necessary to systematically examine selection processes and the consequences of intense selective pressure on subseafloor communities. In sediment from the equatorial Pacific, Indian Ocean and Bering Sea, bacterial taxonomic richness takes a few 100 1000 years to exponentially decline from near-seafloor levels to low values characteristic of deep subseafloor sediment (Walsh et al., 2016). This slow fading of near-seafloor richness is presumably largely due to the disappearance of sedimentary taxa that are well-adapted to near-seafloor conditions. However, it also includes signatures from the water column that are widely dispersed (Hubert et al., 2009; Hamdan et al., 2013; Müller et al., 2014); these mostly disappear within 100,000 to 200,000 years following sediment deposition but can linger as trace constituents for more than a million years (Kirkpatrick et al., 2016). The slowness of the transition from near-seafloor diversity to deep subseafloor diversity is consistent with the report that diversity within four example lineages in Aarhus Bay (Denmark) sediment does not change with sediment age on much shorter timescales (250 to 1750 years ago) (Starnawski et al., 2017). To better understand selection and succession of microbes in marine sediment, we examined community composition of samples that span depositional ages of 660 to 1,300,000 years at two continental-margin sites from very different oceanic regions (Bering Sea and Indian Ocean).

## Materials and Methods

### Site Descriptions and DNA Sampling

**FIGURE 1:**
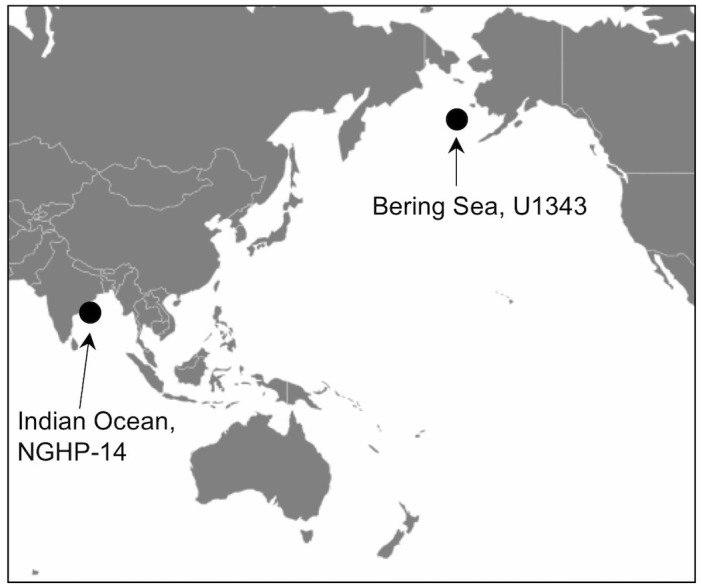
Sampling locations (Bering Sea IODP Site U1343 and Bay of Bengal NGHP Site 14).

We obtained sediment samples from two sites drilled by the *JOIDES Resolution*, one in the Bering Sea and one in the Indian Ocean (Figure 1). Bering Sea Site U1343 was sampled for microbiology as part of Integrated Ocean Drilling Program (IODP) Expedition 323 (Takahashi et al., 2011). Site U1343 is located at 57° 33.4′ N, 175° 49.0′ W, with water depth of 1950 m and seafloor temperature of 2°C. The sediment temperature gradient was 4.9°C/100 m. The site is in the “Green Belt,” a region with very high productivity enhanced by the Bering Sea Current. Mean sedimentation rate is extremely high at U1343 (22–27 cm/kyr) (Takahashi et al., 2011). Sediment at this site was primarily silt with clay, diatoms, and authigenic carbonates in sediment deeper than 35 m below sea floor (mbsf) (Takahashi et al., 2011). Sediment cores were collected with an Advanced Piston Corer (APC). We obtained DNA samples by slicing cores on deck with a sterile spatula, immediately inserting an autoclaved cut-off syringe into the fresh sediment face in the middle of the core, and sealing and freezing the syringe at -80°C. Contamination tracing was conducted onboard using a perfluorocarbon tracer (Takahashi et al., 2011).

Indian Ocean Site 14 (Hole 14A) was cored and sampled during the National Gas Hydrate Program (NGHP) (Collett et al., 2015). Site 14 is located in the Bay of Bengal at 16° 03.6′ N, 82° 05.6′ E in 897 m of water. Bottom water temperature was 7.8°C and the sediment temperature gradient is 3.8°C/100 m (Solomon et al., 2014). Sediment at NGHP-14A is heavily influenced by terrestrial runoff, with total organic carbon (TOC) circa 1.5–2.5% and methane hydrate present (Solomon et al., 2014). Similar to U1343, NGHP-Site 14 cores were obtained with an APC system. Samples for molecular analyses were taken at NGHP-14A by slicing 5 to 10 cm whole core rounds from middle sections of APC cores, using a sterile spatula. The whole rounds were immediately sealed and frozen at -80°C.

### DNA Extraction, Amplification, and Sequencing

We subsampled frozen whole core rounds from NGHP-14A by using a sterile saw blade, thawing cut subsamples, scraping the edges off, and using a sterile spatula to put 0.25 g aliquots of sediment interiors into 2 mL microcentrifuge tubes for DNA extraction. For U1343, we used the same procedure minus the initial saw cut. We extracted DNA using the MO BIO (Carlsbad, CA, United States) PowerLyzer^®^ Powersoil^®^ kit, including optional heating step; the addition of 250 μL phenol-chloroform-isoamyl alcohol (25:24:1, pH 8.0); bead-beating for 90 s on a Biospec (Bartlesville, OK, United States) beadbeater; and a 10 min microcentrifugation after beating. In parallel with sample extractions, we ran duplicate kit blanks with no sediment added. Depending on sediment depth, we pooled between one and six tubes at the end of extraction to enhance yield. We further cleaned extracts with Agencourt (Beckman Coulter, Indianapolis, IN, United States) AMPure XP beads, as per manufacturer’s instructions, and then quantified yield with a Qubit^TM^ (Thermo Fisher Scientific, Waltham, MA, United States) 2.0.

We amplified the v4v5 hypervariable region of the 16S ribosomal gene (rDNA) using primers 518F and 926R for bacteria and 517F and 958R for archaea (Huse et al., 2010), with partial Nextera adapters added for sequencing. We ran reactions in triplicate with 0.4 ng of extracted DNA each, except for the deepest sample from U1343 (300 mbsf) for which we used the maximum possible volume. Controls (tubes without any sediment added) were also extracted, amplified, and sequenced. We used PfuUltra II Fusion HS DNA polymerase, with included kit buffer, added 1× BSA, and 500 nM primers. After 32 cycles of amplification, we pooled triplicate reactions, cleaned again with AMPure XP beads, and transported the extracted DNA to the University of Rhode Island Next Generation Sequencing (NGS) Facility. We sequenced the DNA with Illumina MiSeq, v 3.0 kit chemistry (2 bp × 300 bp paired-end).

### Sequence Analysis

We trimmed and merged de-muliplexed fastq sequence data from Illumina’s Basespace^®^ with CLC Workbench version 6.0 (CLC Bio, Aarhus, Denmark). We used a quality score corresponding to a Phred score of 15 as our trim cutoff, determined empirically to yield the greatest number of successfully merged pairs. After exporting merged reads as a fasta file, we used the Mothur MiSeq pipeline (standard operating procedure at http://www.mothur.org/wiki/MiSeq_SOP; Schloss et al., 2009; Kozich et al., 2013). Before clustering, but after removing problematic reads (e.g., truncated sequences), we subsampled the reads from each sample down to the lowest number of reads obtained for each of four sample sets: Bering Sea (U1343) bacteria and archaea, and Indian Ocean (NGHP-14) bacteria and archaea. We clustered each of these four data sets separately, and also clustered them as combined datasets at the domain level (i.e., all bacterial sequences from both sites were combined, and separately all archaeal sequences from sites were combined). The reference taxonomy was SILVA release 132 (Quast et al., 2012). Inter-site comparisons were done using principal coordinates analysis (PCoA) with Bray–Curtis similarity scores (Bray and Curtis, 1957). PCoA analyses were conducted with mothur (Schloss et al., 2009) based on average-neighbor operational taxonomic unit (OTU) clustering at a 0.03 cutoff level. Singleton and doubleton OTUs were removed. In order to categorize very rare OTUs that may not be detectable in a reproducible fashion due to very low frequency in our data, we used individual OTU tables to determine a “very rare” read count. Below this threshold, random resampling with replacement of the same dataset had a greater than 1% chance of failing to reproduce a given OTU as determined by the formula $P={\left(1-\frac{n}{z}\right)}^{z}$, where n is the number of sequences found for that OTU and z was the total number of sequences (Tables 1, 2).

**Table 1:** Bacterial sequence dataset information.

Site	Depth (mbsf)	Subsampled read count	Total OTU count	OTUs/fraction of reads for:		
				
				Near-seafloor abundant	Near-seafloor moderate	Near-seafloor rare
*U1343*	*0*.*16*	*147,697*	*1,784*	*14/60.0%*	*30/14.7%*	*1,056/23.8%*
U1343	1.66	147,697	1,512	14/60.3%	24/14.5%	409/17.2%
U1343	5.66	147,697	1,369	14/49.2%	27/15.9%	399/20.9%
U1343	8.47	147,697	904	13/52.0%	19/17.4%	253/18.3%
U1343	11.27	147,697	915	13/68.4%	17/10.9%	249/14.6%
U1343	24.1	147,697	652	12/63.7%	14/14.4%	163/6.8%
U1343	86.5	147,697	149	7/68.0%	9/22.6%	36/7.0%
U1343	224.1	147,697	142	9/40.9%	7/30.6%	35/6.8%
U1343	335.3	147,697	192	8/47.1%	9/26.5%	42/8.2%
*NGHP14*	*4*	*131,976*	*2,444*	*18/36.7%*	*46/21.9%*	*1,544/39.1%*
NGHP14	12	131,976	794	13/29.2%	26/22.9%	268/19.5%
NGHP14	21	131,976	621	12/39.1%	18/27.0%	154/22.3%
NGHP14	31	131,976	250	8/24.1%	7/13.6%	46/31.7%
NGHP14	40	131,976	152	6/68.0%	8/16.0%	34/5.4%
NGHP14	49	131,976	183	8/8.0%	8/25.5%	48/55.3%
NGHP14	59	131,976	191	8/13.1%	8/35.7%	56/41.1%
NGHP14	69	131,976	192	7/10.0%	8/40.6%	53/35.0%


*In italics are the “near-seafloor” samples from which we designated which OTUs were abundant, moderate, and rare.*

**Table 2:** Archaeal sequence dataset information.

Site	Depth (mbsf)	Subsampled read count	Total OTU count	OTUs/fraction of reads for:		
				
				Near-seafloor abundant	Near-seafloor moderate	Near-seafloor rare
*U1343*	*0*.*16*	*42,652*	*350*	*10/78.3%*	*23/11.5%*	*103/8.2%*
U1343	1.66	42,652	261	6/81.0%	14/3.2%	41/8.3%
U1343	5.66	42,652	199	6/55.9%	11/15.6%	39/15.1%
U1343	8.47	42,652	123	6/61.9%	13/15.3%	26/14.2%
U1343	11.27	42,652	134	6/61.3%	16/11.6%	30/16.3%
U1343	24.1	42,652	89	5/69.3%	8/4.2%	17/6.1%
U1343	86.5	42,652	18	2/83.8%	1/0.7%	2/4.6%
U1343	224.1	42,652	14	3/2.5%	0/0%	1/12.6%
U1343	335.3	42,652	23	2/8.7%	2/0.1%	1/14.3%
*NGHP14*	*4*	*13,016*	*226*	*11/74.2%*	*26/12.2%*	*41/6.8%*
NGHP14	12	13,016	166	10/20.2%	10/38.0%	14/3.8%
NGHP14	21	13,016	67	8/42.9%	5/18.2%	6/4.9%
NGHP14	31	13,016	82	4/12.4%	3/60.5%	6/0.9%
NGHP14	40	13,016	29	3/15.4%	4/65.0%	1/0.3%
NGHP14	49	13,016	43	4/62.8%	3/7.0%	3/4.8%
NGHP14	59	13,016	49	5/14.7%	3/74.9%	1/0.1%
NGHP14	69	13,016	20	1/38.6%	3/60.4%	1/0.0%


*In italics are the “near-seafloor” samples from which we designated which OTUs were abundant, moderate, and rare.*

**FIGURE 2:**
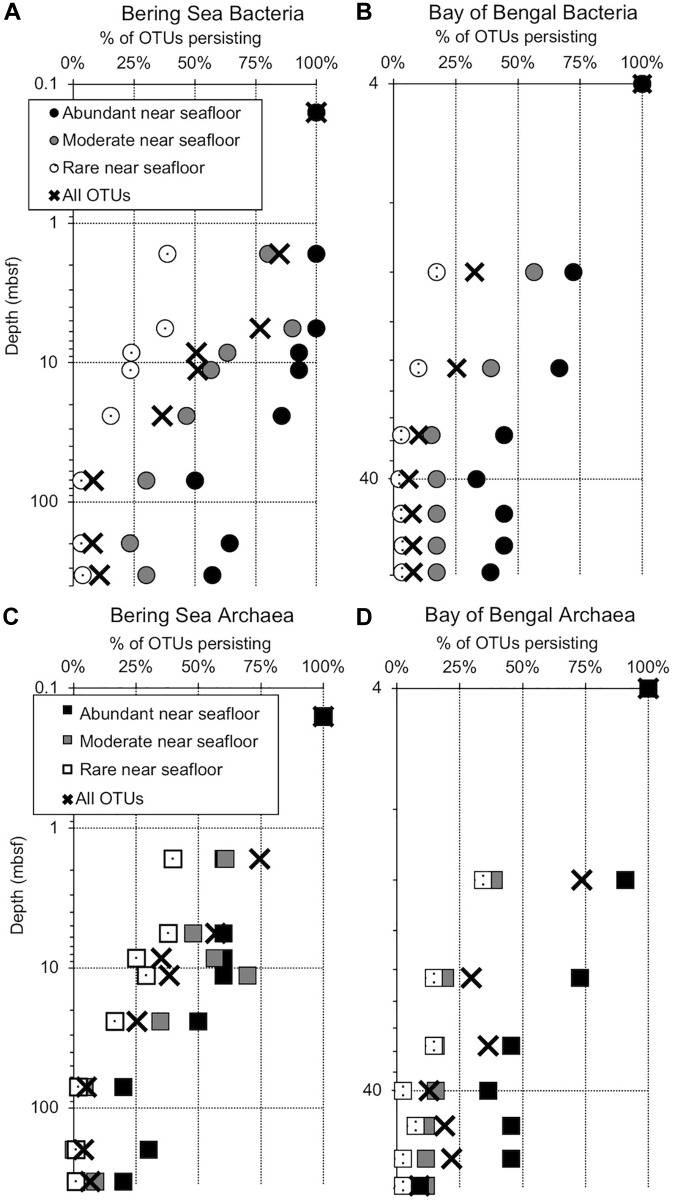
Persistence of abundant, moderate, rare, and total OTUs at depth (meters below seafloor, mbsf), plotted as a percentage of the OTU count for each category in the shallowest sample (“near seafloor”). All categories total 100% for the sample nearest the seafloor, where the number of OTUs in each category is greatest. Circles represent subgroups of bacterial OTUs and squares represent archaea. **(A)** Bering Sea (Site U1343) Bacteria, **(B)** Bay of Bengal (NGHP-14) Bacteria, **(C)** Bering Sea (U1343) Archaea, and **(D)** Bay of Bengal (NGHP-14) Archaea. For **(A,B)**, the shallowest sample is at 0.16 mbsf. For **(C,D)**, the shallowest sample is at 4.0 mbsf.

**FIGURE 3:**
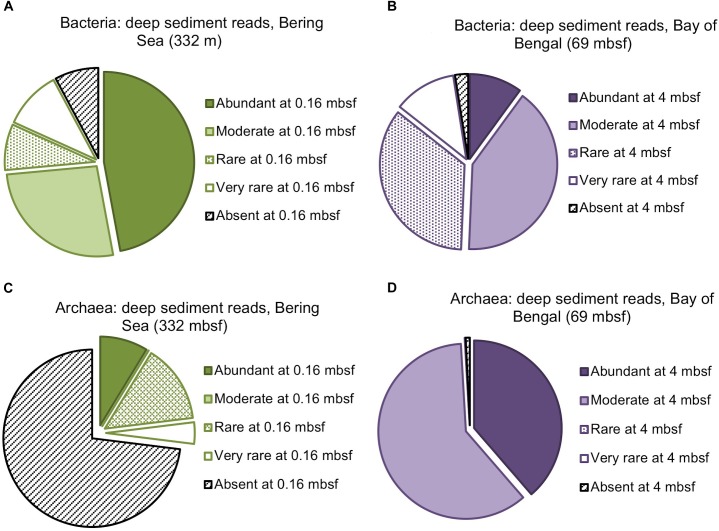
Composition of the deepest sediment communities sampled, as identified by OTU abundance or absence in the shallowest sample from each site. **(A)** Bacteria from 332 mbsf at Bering Sea Site U1343, **(B)** Bacteria from 69 mbsf at Bay of Bengal Site NGHP-14, **(C)** Archaea from 332 mbsf at U1343, **(D)** Archaea at 69 mbsf from NGHP-14.

We assigned all OTUs from the shallowest sample depth for each site (0.16 mbsf at U1343, 4 mbsf at NGHP-14) to one of four categories: abundant (each OTU > 1% of total reads), moderately abundant (0.025% < OTU ≤ 1%), rare (threshold < OTU ≤ 0.025%), and very rare (below threshold) (Figure 2). We give OTU numbers and read counts in Tables 1, 2. These cutoff values are arbitrary but not random, as higher thresholds result in single-digit “abundant” OTUs while lower thresholds cause the read count to become lopsided toward the “abundant” OTUs. We excluded OTUs below this read count threshold from further analysis excepting Figure 3, 4. We also removed OTUs shared between samples and no-sediment controls (kit blanks).

**FIGURE 4:**
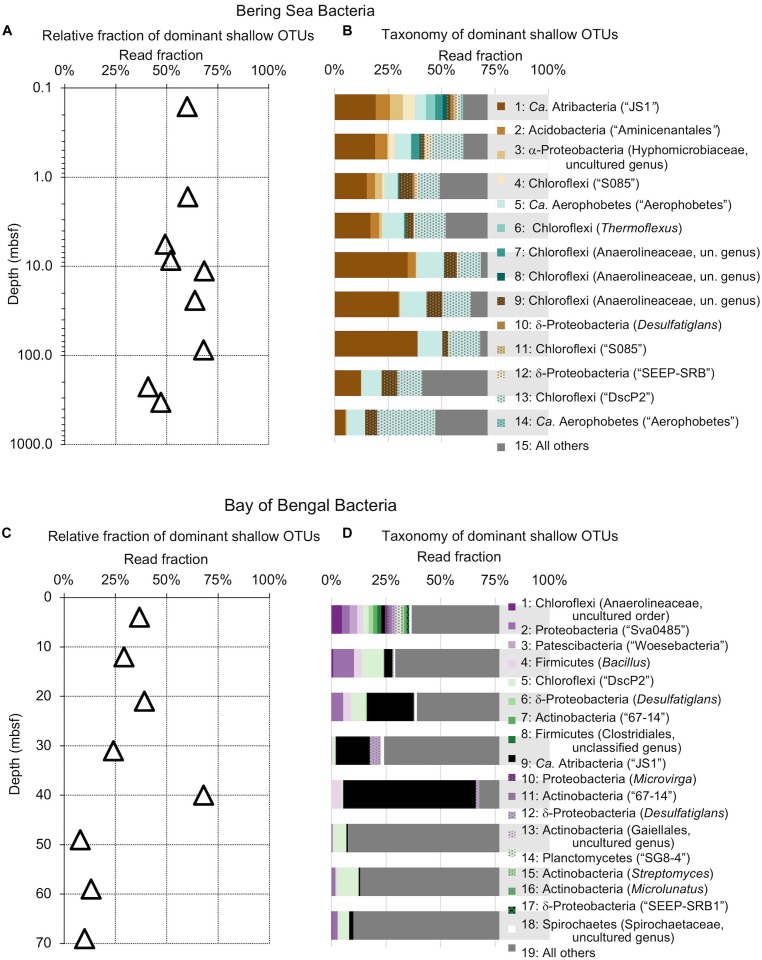
Relative read-based abundance in each sample of bacterial OTUs that are abundant in the shallowest sample at each site. Abundant OTUs are those that comprise more than 1% of the total reads from the shallowest sample. **(A)** For Bering Sea Site U1343, the fraction of total reads represented by all of these OTUs at each sampled depth. **(B)** The fraction of reads represented by each of these OTUs in depth order, with taxonomy. Taxonomic assignments are listed as phylum, and then lowest identifiable level in parentheses (default is genus). “*Ca*.” is Candidatus. For complete taxonomy see Supplemental Tables 1, 2. **(C)** For Indian Ocean Site NGHP-14, the fraction of reads represented by all of these OTUs at each sampled depth. **(D)** The fraction of reads represented by each of these OTUs in depth order, including taxonomy (format same as **B**). Color palette for this figure, Figure 5–9 informed by ColorBrewer 2.0 (Harrower and Brewer, 2003).

Our observed patterns seem unlikely to result from primer bias. There may be taxa that do not extract or amplify with our techniques, or that the given primer sets are biased for or against. While primer bias could result in mis-categorization of OTU abundance, this would obfuscate differences based on abundance. Additionally, as all of the sample datasets presented here are relative to each other, we do not believe that primer bias would produce the consistent trends that we report.

## Results

### Sequencing Data, Richness, and Classification

We amplified bacterial and archaeal 16S v4v5 ribosomal sequences from 9 depths at Integrated Ocean Drilling Program Site U1343 in the Bering Sea (0.16–335 m below seafloor [mbsf]) and 8 depths (4–59 mbsf) at Site 14 of the Indian NGHP in the Bay of Bengal (Figure 1). Sediment ages range to 1.3 Ma at U1343 and 740 kyr at NGHP-14. For Bering Sea Site U1343, we analyzed 147,697 and 42,652 reads per depth after quality control for bacteria and archaea, respectively. For Bay of Bengal Site NGHP-14, we analyzed 131,976 and 13,016 post-quality control reads at each depth for bacteria and archaea, respectively. For both domains and both sites, the total number of OTUs at 97% sequence similarity is highest in the shallowest sample and decreases with depth (Figure 2, “X” markers). Bacterial OTUs at both sites include many reads from Chloroflexi (including Dehalococcoides), Deltaproteobacteria, and *Candidatus* phyla (Atribacteria, Aerophobetes). For bacteria, the deepest sediment samples contain 8–10% of the total OTU richness observed in the shallowest sample (Figure 2A,B). The vast majority of bacterial reads found at depth are represented in the shallowest sample (92.2 and 97.5% for the Bering Sea and Bay of Bengal, respectively) (Figure 3A,B).

For archaea, the deepest sample at each site contains 7–9% of the OTU richness observed in shallow sediment (Figure 2C,D). Archaeal classifications included Euryarchaetoa and Crenarcheota, as well as *Candidatus* phyla (Hydrothermarchaeota, Asgardaeota). A few methanogens are classified. At the Bay of Bengal Site NGHP-14, similar to the bacterial trend, 99% of the archaeal reads found at depth are also present in the shallowest sample from that site (Figure 3D). The pattern is sharply different at Bering Sea Site U1343, where a single Methanosarcinales OTU (*Methermicoccus*) is absent from the shallowest sample but constitutes 68% of all reads at the maximum depth sequenced. Due to the frequency of this OTU in the deepest Bering Sea sample, only 27% of the archaeal reads in the deepest sample match reads present in the near-seafloor community (Figure 3C).

### OTU Categorization and Presence/Absence Downcore

We separated the reads from the shallowest, most OTU-rich dataset for each domain at each site into three broad categories: abundant (each OTU > 1% of reads), moderately abundant (≤1% but >0.25%), and rare (≤0.25% but above a minimum threshold value; see section “Materials and Methods”) (Tables 1, 2). In all cases, more OTUs occur in the rare fraction than in the other two fractions. However, the relatively small number of OTUs in the abundant fraction accounts for 37–60% of bacterial and 74–78% of archaeal reads in the shallowest sample.

Operational taxonomic units represented by abundant reads in the near-seafloor sediment appear at greater depths much more frequently than OTUs assigned to other categories (Figure 2). In the Bering Sea, 57 and 20% of the bacterial and archaeal OTUs assigned to the abundant category, respectively, are still present at the greatest depth sampled (Figure 2A,B). In contrast, only 4 and 1%, respectively, of OTUs assigned to the rare category occur in the deepest sediment (Figure 2A,B). OTUs from the moderately abundant category appear at depth less frequently than abundant OTUs, but more frequently than rare OTUs. In the Bay of Bengal, 39 and 9%, respectively, of bacterial and archaeal OTUs assigned to the abundant category are present at the greatest depth (Figure 2C,D). Only 3 and 2%, respectively, of bacterial and archaeal OTUs assigned to the rare category are present in the deepest sediment at this site.

We also categorized a fourth category in the shallowest sample, labeled “very rare,” which falls below a random resampling threshold and thus may “appear” or “disappear” in samples by chance, even as its underlying frequency does not change. In the near-seafloor sediment, this category constitutes a small fraction of our data, consisting of 1.5 and 2.0% of bacterial and archaeal reads in the Bering Sea and 2.4 and 6.8% in the Bay of Bengal. Nonetheless, at depth, the OTUs classified into this group based on the shallowest sample account for 10–11% of bacterial reads, and 0.1–4% of archaeal reads.

### Taxonomic and Depth Trends

**FIGURE 5:**
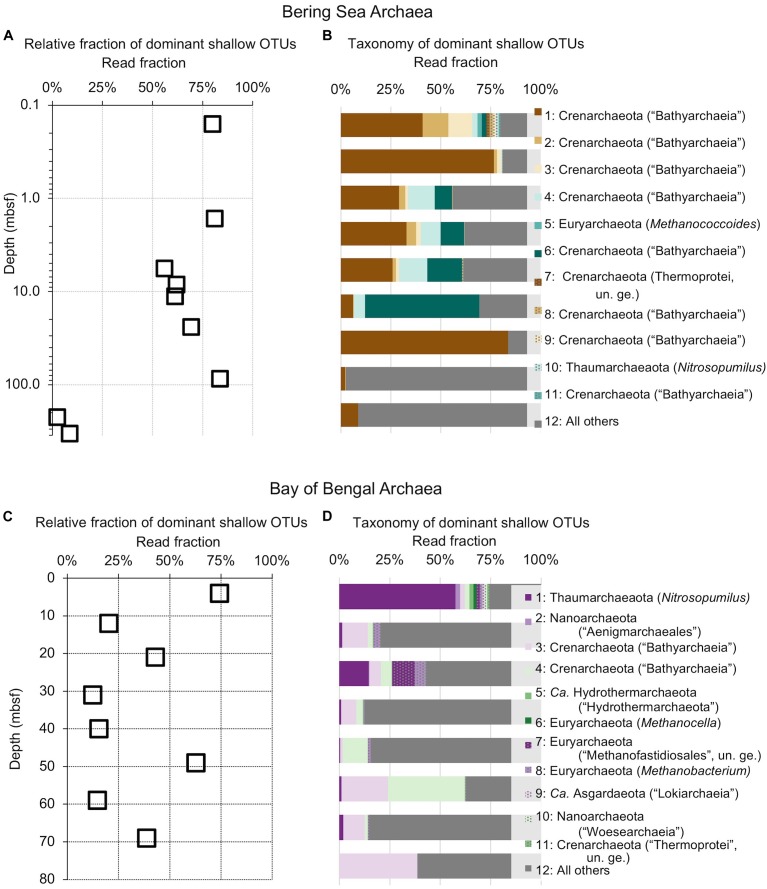
Relative read-based abundance in each sample of archaeal OTUs that are abundant in the shallowest sample at each site. Abundant OTUs are those that comprise more than 1% of the total reads from the shallowest sample. **(A)** For Bering Sea Site U1343, the fraction of total reads represented by all of these OTUs at each sampled depth. **(B)** The fraction of reads represented by each of these OTUs in depth order, with taxonomy. Taxonomic assignments are listed as phylum, and then lowest identifiable level in parentheses (default is genus). “*Ca*.” is Candidatus, “un. ge.” is uncultured genus. For complete taxonomy see Supplementary Tables 1, 2. **(C)** For Indian Ocean Site NGHP-14, the fraction of reads represented by all of these OTUs at each sampled depth. **(D)** The fraction of reads represented by each of these OTUs in depth order, including taxonomy (format same as **B**).

The taxonomic assignments of our near-seafloor abundant OTUs vary between sites, but are broadly consistent with previous studies of the marine sedimentary deep biosphere (Briggs et al., 2012; Mills et al., 2012; Walsh et al., 2015; Petro et al., 2017). Many bacterial and archaeal OTUs classify with environmental clones. Bacterial OTUs included Chloroflexi, Deltaproteobacteria, and Actinobacteria (Figure 4). Archaeal OTUs include Crenarcheota and Euryarcheota, including some known methanogens, and *Candidatus* phyla Hydrothermarchaeota and Asgardaeota (Figure 5). As a group, the OTUs that are abundant in the near-seafloor sediment generally account for a smaller fraction of sequences at depth (Figure 4, 5).

**FIGURE 6:**
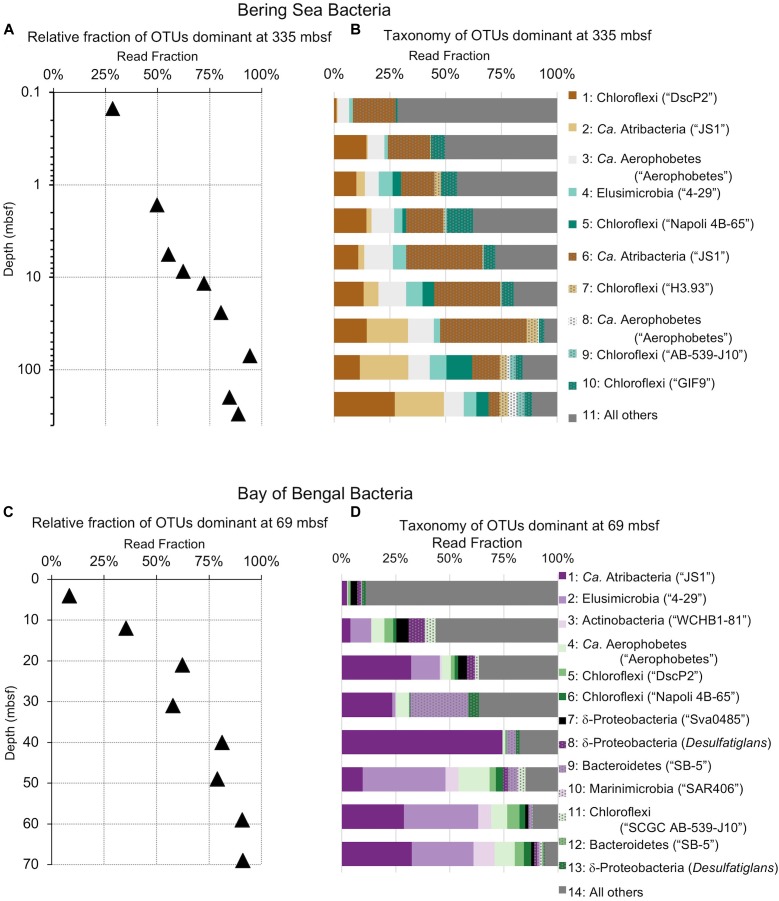
Relative read-based abundance in each horizon of bacterial taxa that dominate the deepest sample. Deep dominant OTUs are OTUs represented by >1% of the sequences returned from the deepest sample. **(A)** The fraction of bacterial reads comprised by all of the deep dominant OTUs at each depth for Bering Sea site U1343. **(B)** The fraction of reads represented by each of these OTUs in depth order, with taxonomy. Taxonomic assignments are listed as phylum, and then lowest identifiable level in parentheses (default is genus). “*Ca*.” is Candidatus. For complete taxonomy see Supplementary Tables 1, 2. **(C)** The fraction of bacterial reads comprised by all of the deep dominant OTUs at each depth for Bay of Bengal site NGHP-14. **(D)** The fraction of reads represented by each of these OTUs in depth order, including taxonomy.

**FIGURE 7:**
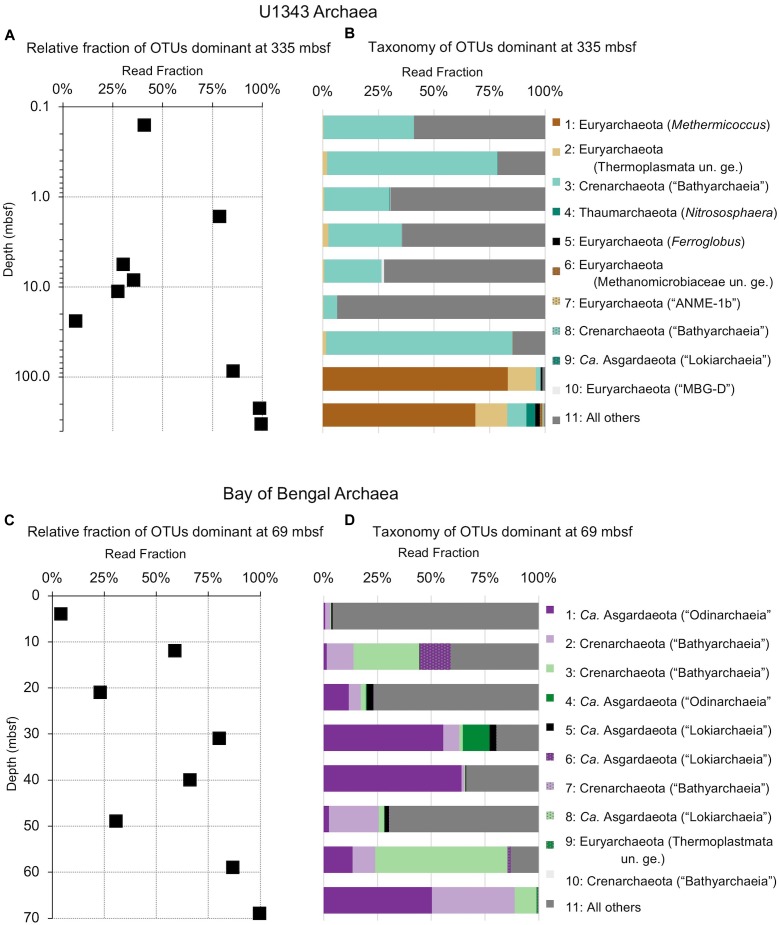
Relative read-based abundance in each horizon of archaeal taxa that dominate the deepest sample. Deep dominant OTUs are OTUs represented by >1% of the sequences returned from the deepest sample. **(A)** The fraction of archaeal reads comprised by all of the deep dominant OTUs at each depth for Bering Sea site U1343. **(B)** Archaeal taxonomy and relative abundance in depth order. Taxonomic assignments are listed as phylum, and then lowest identifiable level in parentheses (default is genus). “*Ca*.” is Candidatus, “un. ge.” is uncultured genus. For complete taxonomy see Supplementary Tables 1, 2. **(C)** The fraction of archaeal reads comprised by all of the deep dominant OTUs at each depth for Bay of Bengal site NGHP-14. **(D)** Archaeal taxonomy and relative abundance in depth order. Format same as **(B)**.

**FIGURE 8:**
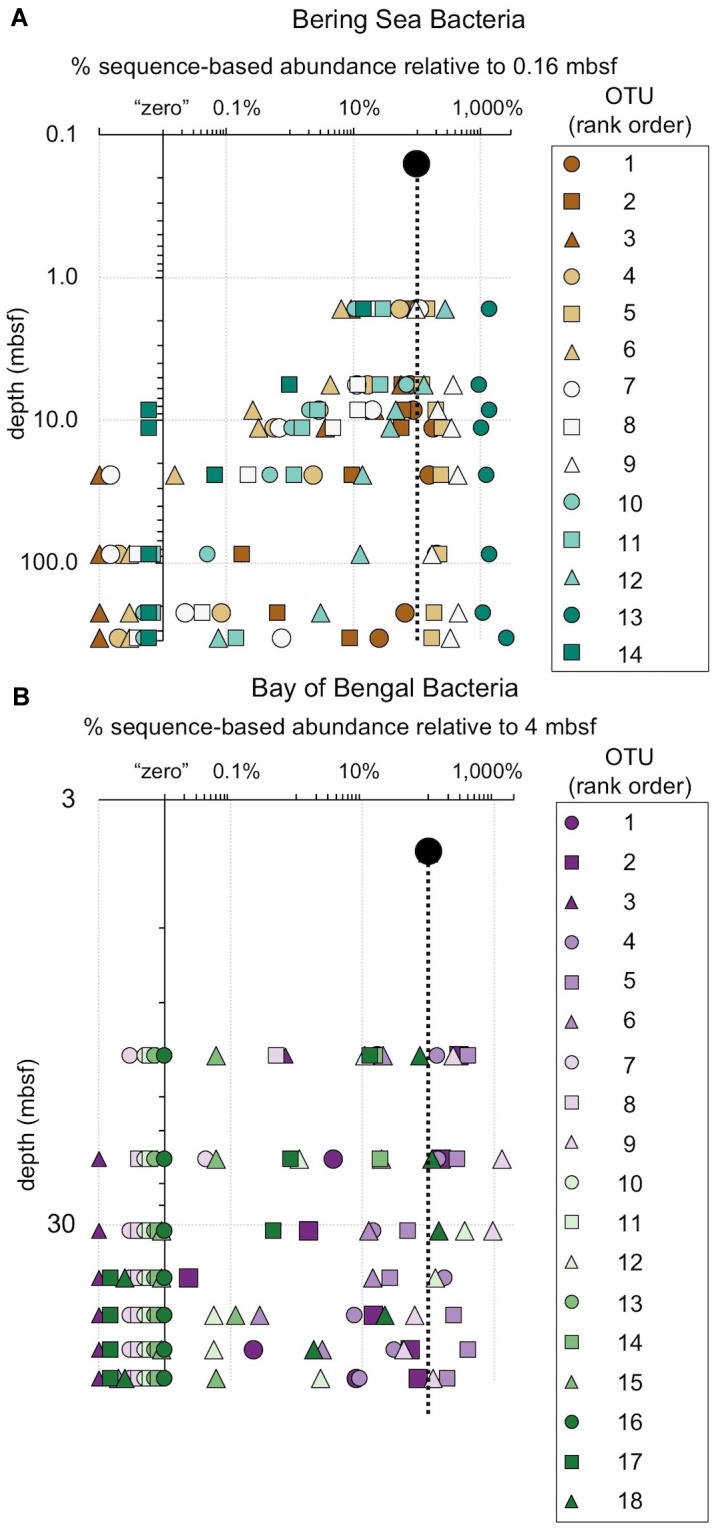
The relative fraction of reads at each depth assigned to individual bacterial OTUs (97% similarity cutoff) that are abundant in the shallowest sample at **(A)** Bering Sea Site U1343 and **(B)** Bay of Bengal Site NGHP-14. The dashed line represents 100% of surface reads (i.e., no change in relative abundance with depth). Values to the right of the dashed line indicate higher frequency of the sequences assigned to each OTU (relative to the shallowest sample), whereas values to the left indicate lower frequency relative to the shallowest sample. Rank order is the same as Figure 4. For reference, for **(A)** rank order 5 is *Ca.* “Aerophobetes,” 9 is Chloroflexi (Anaerolinaceae), and 13 is Chloroflexi (Dehalococcoidia; “DscP2”). For **(B)** rank order 5 is Chloroflexi (Dehalococcoidia; “DscP2”) and 9 is *Ca.* “Atribacteria” (JS1). Axes are logarithmic. Values to the left of the y-axis represent “zero” (not detected).

**FIGURE 9:**
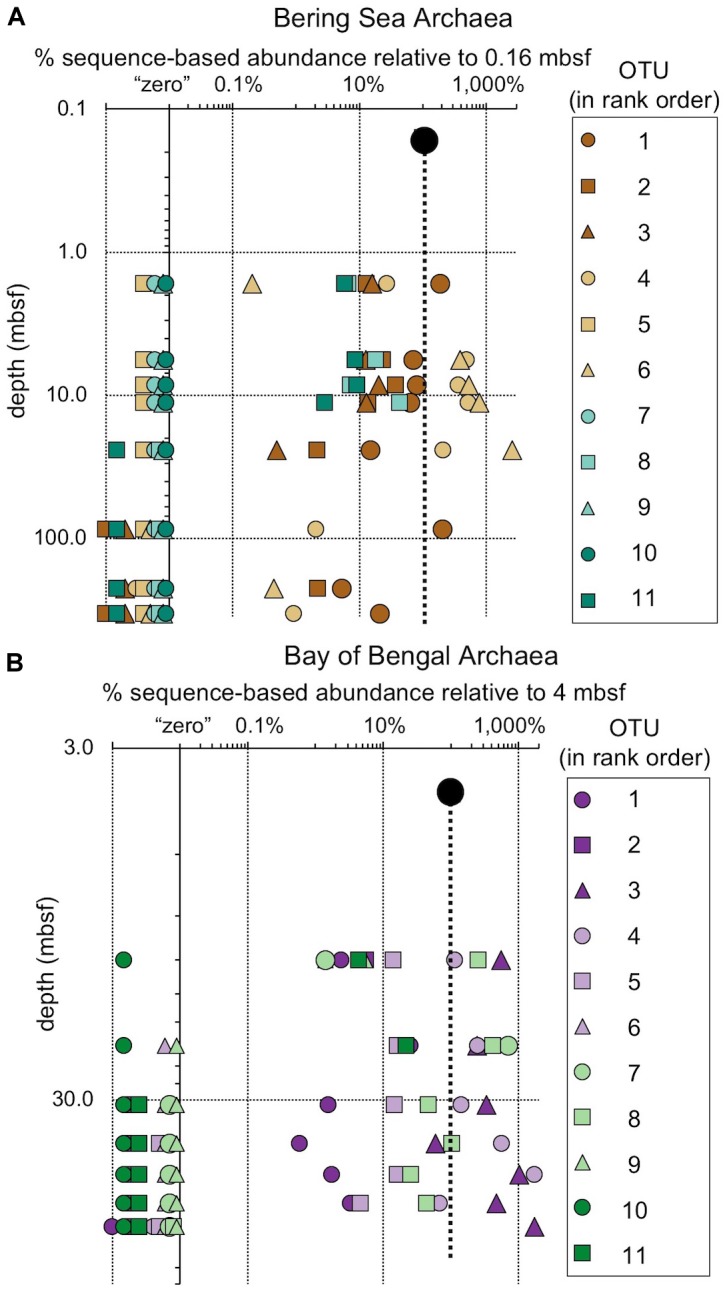
The relative fraction of reads assigned to individual archaeal OTUs (97% similarity cutoff) that are abundant in the shallowest sample at **(A)** Bering Sea Site U1343 and **(B)** Bay of Bengal Site NGHP-14. The dashed line represents 100% of surface reads (no change in relative abundance with depth). Values to the right of the dashed line indicate higher frequency of the sequences assigned to each OTU (relative to the shallowest sample), whereas values to the left indicate lower frequency relative to the shallowest sample. Rank order same as Figure 5. For reference, for **(A)** rank order 1 is “Bathyarchaeia,” and 4 and 6 also fall in this group. For **(B)** rank order 3 is in “Bathyarchaeia” and so is 4. Axes are logarithmic. Values to the left of the y-axis represent “zero” (not detected).

**FIGURE 10:**
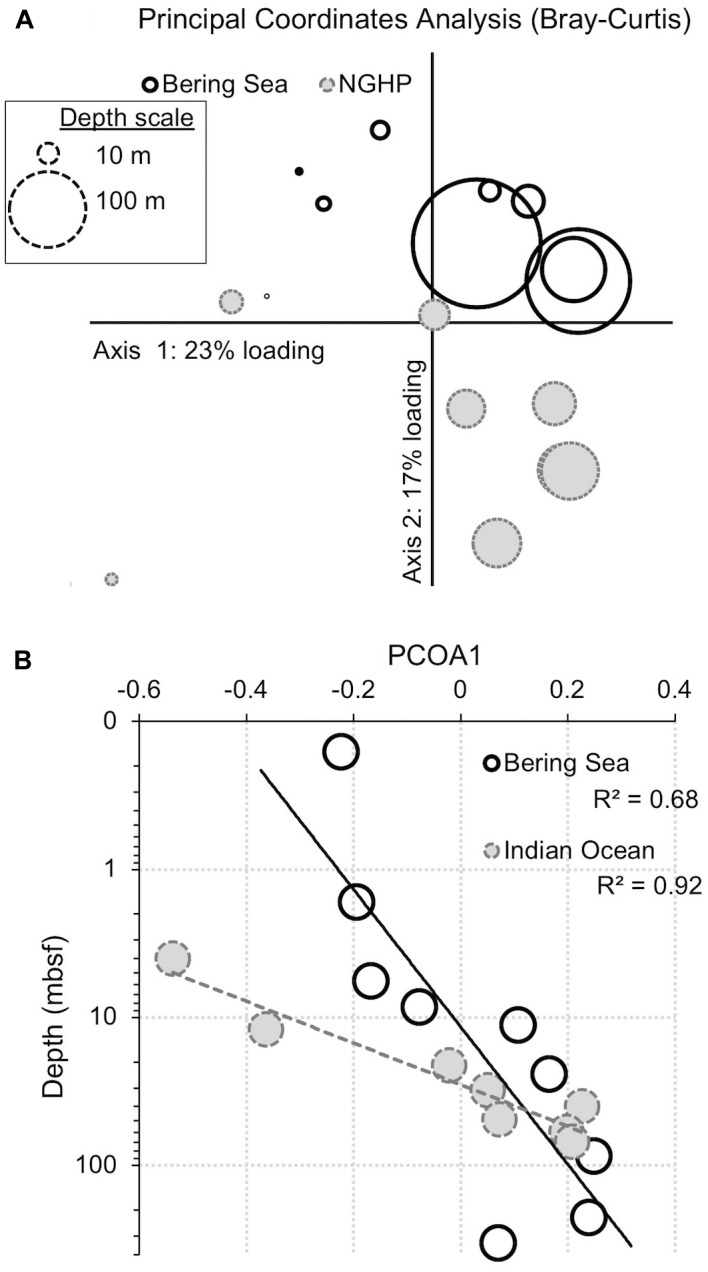
Principal coordinates analysis (PCoA) for bacteria showing the similarity of samples from the two different sites and different depths. Similarity was determined by abundance-weighted metrics (Bray–Curtis). **(A)** PCoA plot. The loading explained by the two axes is shown (23% for x, 17% for y). Symbol size represents sample depth. **(B)** Primary axis ordination (23% of variance) versus sample depth, with an exponential fit. Open symbols with solid outlines indicate Bering Sea samples. Gray symbols with dashed outlines indicate Bay of Bengal samples.

The deepest samples at our two sites are dominated by a small number of OTUs for both domains (Figure 6, 7). Consistent with the overall loss of richness, the 10 OTUs most frequently observed in each of our deepest samples account for 89–99% of sequences in those samples. These same OTUs also generally occur in the shallow sediment, but only account for 4–41% of the near-seafloor archaeal sequences and 8–28% of the near-seafloor bacterial sequences. Conversely, the majority of near-seafloor OTUs decrease in relative frequency with depth (Figure 8, 9). Comparisons in overall bacterial community structure between depths and sites show separation between the two locations (Figure 10A) and also correlation between the primary PCoA axis and depth below seafloor (Figure 10B).

## Discussion

Cell counts and biomass generally decrease exponentially with depth in marine sediment (Kallmeyer et al., 2012). The deep marine sedimentary ecosystem is energy-limited, with some of the lowest biomass turnover and reaction rates known on Earth (D’Hondt et al., 2002; Jørgensen and D’Hondt, 2006; Jørgensen and Boetius, 2007; Lomstein et al., 2012; Røy et al., 2012; D’Hondt et al., 2014; Lever et al., 2015; Jørgensen and Marshall, 2016). Previous comparisons of multiple sites indicate that (i) bacterial diversity declines in parallel with total organic respiration, as a function of sediment age (Walsh et al., 2016), and (ii) cell abundance declines as a function of sediment age (Kallmeyer et al., 2012; Jørgensen and Marshall, 2016). The coupling of energy limitation with these overall decreases in microbial community size and diversity imply intense selection in this environment (Petro et al., 2017).

### Preferential Persistence of OTUs Abundant in Shallow Sediment

At both sites, both bacterial and archaeal communities decrease in OTU richness with sediment depth, similar to previous studies (Walsh et al., 2015; Walsh et al., 2016). For both domains at both sites, the observed number of OTUs exponentially declines by 90% or more from the seafloor to relatively stable values at depths greater than a few 10s of meters in Bay of Bengal sediment and nearly 100 m in the Bering Sea sediment (Figure 2). This exponential decrease implies that culling of OTUs is most intense when the sediment is relatively young (in the first few 10 to 100s of kyrs after sediment deposition).

As described in our results, sequences that are most prevalent in shallow sediment are most likely to be detected at greater depths. Sequences that are less common in the shallow sediment (moderately abundant) are present with lower frequency at depth compared to the most common sequences, and sequences that are somewhat rare in the shallow sediment are almost entirely absent from the greatest depths (Figure 2). These results are consistent with persistence of OTUs that were initially seeded by a shallow sedimentary community that has been relatively consistent in composition over the past 0.74 Ma (NGHP-14) to 1.3 Ma (U1343).

The preferential persistence suggests that the influence of primary selection processes in shallow sediment continues into deep sediment. While distinct geochemical transitions occur in the subseafloor sediment of these sites (Wehrmann et al., 2011; Solomon et al., 2014), overall community composition appears primarily controlled by composition of the community that initially colonized the sediment. The extent to which this pattern results from the success of a generalist lifestyle and/or basic “indifference” to the changing geochemical regimes in which they reside remains to be seen.

This preferential persistence is not an artifact of random culling (equal probabilities of mortality) within the community. If the population was culled randomly, the community would shrink but the relative proportions of abundant, moderately abundant and rare OTUs in our extracts and sequence datasets would remain unchanged. Because we normalized the extracted DNA input prior to amplification and subsequently subsampled the sequencing results to equal numbers of reads per sample, random culling should lead to the same relative abundances of OTUs throughout the sediment column.

### Most Taxa That Persist Are Selected Against

While one might perceive OTUs that persist to depth as successful, most decrease in relative frequency with depth. Only a small number (0–3) of the OTUs abundant near the seafloor increase in relative frequency with depth (Figure 8, 9). For example, at each site, one bacterial OTU assigned to Dehalococcoidia increases in relative frequency with depth. The majority of abundant OTUs in the near-seafloor sediment persist to depth, but decrease in their relative frequency in the sequence data. Other OTUs abundant in near-seafloor sediment decline with increasing depth and ultimately disappear.

### Local Opportunists or Widespread Subseafloor Specialists?

When we use principal coordinate analysis to compare bacterial communities, the dominant factor (PCoA1) explains 23% of the variance (Figure 10A). This factor correlates with sediment age (*R*^2^ = 0.68 and 0.92 for the Bering Sea and Indian Ocean, respectively; Figure 10B). This factor shows that some of the change in 97%-similar bacterial OTU dominance with sediment depth and age is parallel at these two very distant sites. When we use principal coordinate analysis to compare archaeal communities, the results are similar, but noisier, with fewer OTUs (Table 2) and with less variance explained by PCoA1 and PCoA2 (18 and 15%, respectively; Supplementary Figure 1). The archaeal PCoA2 correlates modestly with depth (*R*^2^ = 0.74 and 0.34 for the Bering Sea and Indian Ocean, respectively), showing that a small amount of the change in 97%-similar archaeal OTU dominance with sediment depth and age is also parallel at these two sites. The archaeal PCoA1 does not correlate with depth (Supplementary Figure 1B). Furthermore, for both Bacteria and Archaea, some of the OTUs that predominate at depth at each individual site cluster together at higher taxonomic levels (e.g., *Ca*. Atribacteria or Chloroflexi; Dehalococcoidia) (Supplementary Tables 1, 2) This result is consistent with previous demonstrations that relatively abundant microorganisms in similar subseafloor environments of geographically distant sites often belong to the same phyla or orders (Inagaki et al., 2006; Petro et al., 2017).

Despite this partial similarity in bacterial and archaeal community change with depth, many of the OTUs that dominate at depth are different at the two sites (Figure 6, 7, 10). In the principal coordinate plots, this difference is most clearly illustrated by the bacterial communities; the Bray–Curtis abundance-weighted similarity of the sequenced bacterial communities from all of the depths at both sites shows that (i) the deep samples from each site are most similar to other deep samples from that site, and (ii) Bering Sea samples group separately from Indian Ocean samples (Figure 10A). As these two locations harbor different communities at the seafloor, this result is consistent with the deep community of each site being a subset of the local seafloor community, rather than a grown-in group of geographically widespread subseafloor specialists.

### Do Deep Subseafloor Microbes Replicate?

The extraordinarily low per-cell energy fluxes in subseafloor sediment appear barely sufficient for molecular repair of living biomass, leading to speculation that subseafloor cells reproduce rarely, if at all (D’Hondt et al., 2014; Jørgensen and Marshall, 2016). Consequently, we ask whether the organisms represented by OTUs commonly found at depth have to replicate to achieve their relative dominance of the DNA pool, or if they simply disappear more slowly (i.e., exhibit reduced mortality rates).

The vast majority of sequences found in the deepest sediment (aged 1.3 Ma at Bering Sea Site U1343 and 740 kyr at Indian Ocean Site NGHP-14) are also present near the seafloor (Figure 3). Although overall richness drops with age, a few bacterial OTUs migrate from relatively rare in our sequence datasets to relatively abundant with increasing sediment depth (Figure 3, 6, 7). For Archaea at these sites as well, the majority of OTUs found in deep sediment are also present but relatively rare in the shallowest (youngest) sediment. The only notable exception occurs at U1343 in the Bering Sea, where a single OTU assigned to Methanosarcinales dominates the archaeal reads at the greatest depths but is absent from the sequence dataset of our shallowest sample. This result, that most sequences found in 0.74- to 1.3-Ma sediment are present as relatively rare sequences near the seafloor, greatly extends the recent discovery that the dominant OTUs in 2000-ka sediment of Aarhus Bay (Denmark) are a small subset of the OTUs present at the Aarhus seafloor (Starnawski et al., 2017).

**FIGURE 11:**
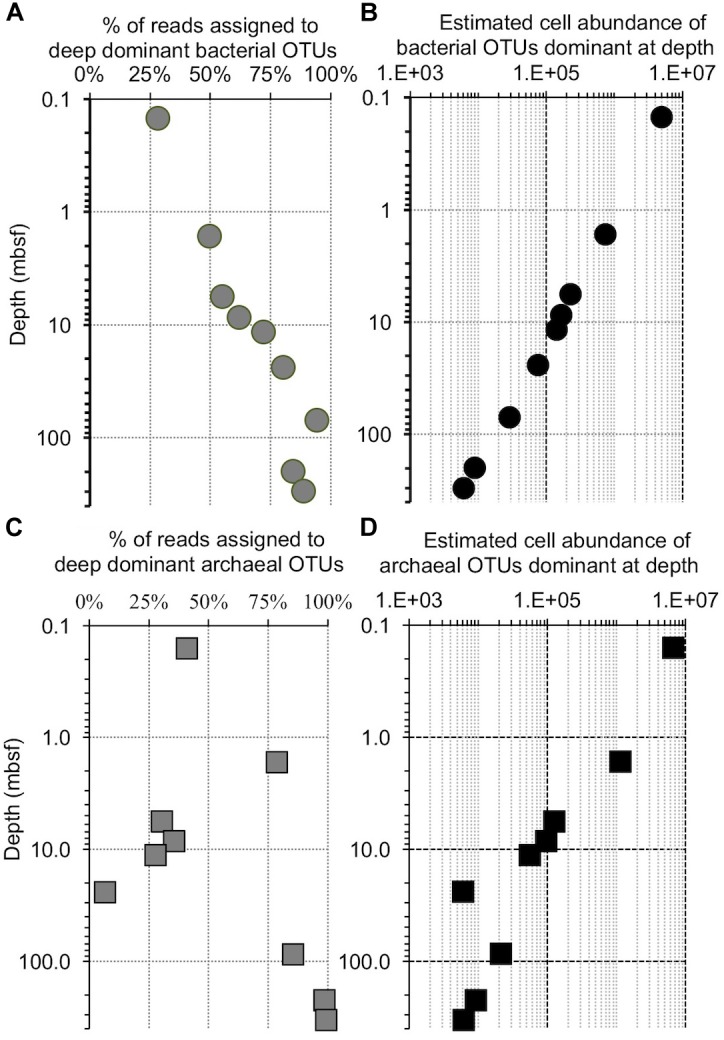
Comparison of the increase in OTU dominance to the decrease in cell abundance with increasing depth at Bering Sea Site U1343. **(A)** The fraction of reads contained in a bin of the 10 bacterial OTUs that are most abundant in the deepest samples at Bering Sea (Site U1343). **(B)** The calculated number of cells represented by these OTUs versus depth (logarithmic scale), determined from **(A)** and the trendline of U1343 cell counts with depth (cell counts from Kallmeyer et al., 2012). **(C)** The fraction of reads contained in a bin of the 10 archaeal OTUs that are most abundant in the deepest sample at Site U1343. **(D)** Similar to **(B)**, the calculated number of cells represented by these OTUs versus depth (logarithmic scale).

Previously published cell counts from Bering Sea site U1343 (Kallmeyer et al., 2012) allow us to quantitatively test if replication is necessary to dominate the deep subseafloor community. The decrease in cell numbers with depth at this site overshadows the observed increase in relative sequence dominance by 2 to 3 orders of magnitude, assuming a constant ratio of sequences to cells, and of bacteria to archaea (Figure 11). This result indicates that all of the bacterial taxa decline in absolute concentration, and replication is not necessary to dominate the bacterial community at depth. In other words, for all taxa, mortality exceeds replication.

As described above, our U1343 archaeal data include one sequence (Methanosarcinales; *Methermicoccus* type) that is relatively abundant at depth but absent from our near-seafloor reads. Furthermore, unlike for the bacteria, some archaeal sequences grouped into specified OTUs wax and wane with depth at U1343 (Figure 7, 11). Consequently, at least this one OTU may differ from the co-occurring microbial populations in either (i) varying more over time in its initial near-seafloor abundance or (ii) being more dynamic in the deep subseafloor (e.g., exhibiting growth or varying in its relative rate of mortality with sediment age or depth). However, given the sequence abundance of this OTU at 220 and 332 mbsf and the total number of reads per sample, it is possible that this OTU was present in the near-seafloor DNA pool at a level below detection (i.e., less than 3 out of 147,697), but rose to relative dominance at depth without replication. In short, like the predominant subseafloor bacterial OTUs, this archaeal OTU could have become a majority subseafloor OTU simply due to the degradation and disappearance of other archaeal phylotypes (Figure 11). Similarly, other taxa that are less abundant in the deep sediment and not detected in the near-seafloor sediment may be present but below our detection limit in the near-seafloor sediment, such that deeper sequencing (greater number of reads per sample) might reveal 100% of the deep-subseafloor taxa to be present in the near-seafloor sediment.

## Conclusion

The best predictor of persisting taxa over 100s of 1000s to more than a million years is relative sequence abundance in the youngest sediment. The two locations harbor different communities at the seafloor. The OTUs that dominate at depth comprise a subset of the local seafloor community at each site, rather than a grown-in group of geographically widespread subseafloor specialists. This result suggests that many aspects of subseafloor community structure are set in near-seafloor sediment, with limited roles for selection and/or specialization in the energy-limited sediment below. Comparison to previously published cell counts indicates that all of the bacterial taxa at the Bering Sea site decline in absolute concentration with sediment depth, and replication is not necessary to dominate the bacterial community at depth. The first-order pattern is broadly similar for Archaea, although relative abundance of the dominant archaeal taxon waxes and wanes from sample to sample. This broad pattern, of mortality exceeding replication, suggests that investigation of the causes of reduced morbidity may be key to understanding the composition of subseafloor sedimentary microbial communities over million-year timescales.

## Author Contributions

SD’H and EW designed the sampling plan for the Bering Sea. EW conducted Bering Sea field work. JK processed sediment samples, extracted and amplified DNA, and prepared samples for sequencing. JK analyzed data, with input from EW and SD’H. JK prepared figures and manuscript, with edits and revisions from SD.

## Conflict of Interest Statement

The authors declare that the research was conducted in the absence of any commercial or financial relationships that could be construed as a potential conflict of interest.
