# Supplementary material for: Microbial Selection and Survival in Subseafloor Sediment
Source: Front Microbiol. 2019 May 14;10:956. doi: 10.3389/fmicb.2019.00956 (PMC6527604; doi:10.3389/fmicb.2019.00956)
Supplement: Supplementary file 1 [file Data_Sheet_1.pdf]

Supplementary Material

Article Title

John B. Kirkpatrick*, Emily A. Walsh, Steven D'Hondt

*** Correspondence:** Corresponding Author: john.b.kirkpatrick@gmail.com

1 Supplementary Figures and Tables

Supplementary Table 1. Abundant archaeal sequence types, in rank order, for Bering Sea (BS) Site U1343 and Bay of Bengal Site NGHP-14 (BB). Taxonomic assignment, based on SILVA release 132, is listed down to genus level. Identical taxonomic assignments at a given site indicates similar taxonomy for unique, non-identical OTUs. For archaea, asterisk (*) indicates highest taxonomic level where nomenclature uncertainty or Candidatus status may apply; this is unrelated to certainty of taxonomic assignment.

OTU ID (rank order)	Location	Taxonomic assignment	Location	Taxonomic assignment
1	BS	Crenarchaeota; *Bathyarchaeia; Bathyarchaeia; Bathyarchaeia; Bathyarchaeia	BB	Thaumarchaeota; Nitrososphaeria; Nitrosopumilales; Nitrosopumilaceae; *Nitrosopumilus*;
2	BS	Crenarchaeota; *Bathyarchaeia; Bathyarchaeia; Bathyarchaeia; Bathyarchaeia	BB	Nanoarchaeaeota; *Nanohaloarchaeia; Aenigmarchaeales; Aenigmarchaeales; Aenigmarchaeales
3	BS	Crenarchaeota; *Bathyarchaeia; Bathyarchaeia; Bathyarchaeia; Bathyarchaeia	BB	Crenarchaeota; *Bathyarchaeia; Bathyarchaeia; Bathyarchaeia; Bathyarchaeia
4	BS	Crenarchaeota; *Bathyarchaeia; Bathyarchaeia; Bathyarchaeia; Bathyarchaeia	BB	Crenarchaeota; *Bathyarchaeia; Bathyarchaeia; Bathyarchaeia; Bathyarchaeia

5	BS	Euryarchaeota; Methanomicrobia; Methanosarcinales; Methanosarcinaceae; *Methanococcoides*	BB	*Hydrothermarchaeota; Hydrothermarchaeota; Hydrothermarchaeota; Hydrothermarchaeota; Hydrothermarchaeota
6	BS	Crenarchaeota; *Bathyarchaeia; Bathyarchaeia; Bathyarchaeia; Bathyarchaeia	BB	Euryarchaeota; Methanomicrobia; Methanocellales; Methanocellaceae; *Methanocella*
7	BS	Crenarchaeota; Thermoprotei; *uncultured; uncultured; uncultured	BB	Euryarchaeota; Thermococci; *Methanofastidiosales; uncultured; uncultured
8	BS	Crenarchaeota; *Bathyarchaeia; Bathyarchaeia; Bathyarchaeia; Bathyarchaeia	BB	Euryarchaeota; Methanobacteria; Methanobacteriales; Methanobacteriaceae; *Methanobacterium*
9	BS	Crenarchaeota; *Bathyarchaeia; Bathyarchaeia; Bathyarchaeia; Bathyarchaeia	BB	*Asgardaeota; Lokiarchaeia; Lokiarchaeia; Lokiarchaeia; Lokiarchaeia
10	BS	Thaumarchaeota; Nitrososphaeria; Nitrosopumilales; Nitrosopumilaceae; *Nitrosopumilus*	BB	Nanoarchaeaeota; *Woesearchaeia; Woesearchaeia; Woesearchaeia; Woesearchaeia
11	BS	Crenarchaeota; *Bathyarchaeia; Bathyarchaeia; Bathyarchaeia; Bathyarchaeia	BB	Crenarchaeota; Thermoprotei; *uncultured; uncultured; uncultured

Supplementary Table 2. Abundant bacterial sequence types, in rank order, for Bering Sea Site U1343 (BS) and Bay of Bengal Site NGHP-14 (BB). Taxonomic assignment, based on SILVA release 132, is listed down to genus level. Identical taxonomic assignments at a given site indicates similar taxonomy for unique, non-identical OTUs.

**OTU ID (rank order)**	**Location**	**Taxonomic assignment**	**Location**	**Taxonomic assignment**
1	BS	Candidatus Atribacteria; JS1; JS1; JS1; JS1	BB	Chloroflexi; Anaerolineae; uncultured; uncultured; uncultured
2	BS	Acidobacteria; Candidatus Aminicenantia; Aminicenantales; Aminicenantales; Aminicenantales	BB	Proteobacteria; Deltaproteobacteria; Sva0485; Sva0485; Sva0485
3	BS	Proteobacteria; Alphaproteobacteria; Rhizobiales; Hyphomicrobiaceae; uncultured	BB	Patescibacteria; Candidatus Microgenomatia; Woesebacteria; Woesebacteria; "Woesebacteria"
4	BS	Chloroflexi; Dehalococcoidia; S085; S085; S085	BB	Firmicutes; Bacilli; Bacillales; Bacillaceae; *Bacillus*
5	BS	Candidatus Aerophobetes; Aerophobetes; Aerophobetes; Aerophobetes; Aerophobetes	BB	Chloroflexi; Dehalococcoidia; DscP2; DscP2; DscP2
6	BS	Chloroflexi; Anaerolineae; Thermoflexales; Thermoflexaceae; *Thermoflexus*	BB	Proteobacteria; Deltaproteobacteria; Desulfarculales; Desulfarculaceae; *Desulfatiglans*
7	BS	Chloroflexi; Anaerolineae; Anaerolineales; Anaerolineaceae; uncultured	BB	Actinobacteria; Thermoleophilia; Solirubrobacterales; 67-14; 67-14
8	BS	Chloroflexi; Anaerolineae; Anaerolineales; Anaerolineaceae; uncultured	BB	Firmicutes; Clostridia; Clostridiales;

				Ruminococcaceae; unclassified
9	BS	Chloroflexi; Anaerolineae; Anaerolineales; Anaerolineaceae; uncultured	BB	Candidatus Atribacteria; JS1; JS1; JS1; JS1
10	BS	Proteobacteria; Deltaproteobacteria; Desulfarculales; Desulfarculaceae; *Desulfatiglans*	BB	Proteobacteria; Alphaproteobacteria; Rhizobiales; Beijerinckiaceae; *Microvirga*
11	BS	Chloroflexi; Dehalococcoidia; S085;S085; S085	BB	Actinobacteria; Thermoleophilia; Solirubrobacterales; 67-14; 67-14
12	BS	Proteobacteria; Deltaproteobacteria; Desulfobacterales; Desulfobacteraceae; SEEP-SRB	BB	Proteobacteria; Deltaproteobacteria; Desulfarculales; Desulfarculaceae; *Desulfatiglans*
13	BS	Chloroflexi; Dehalococcoidia; DscP2; DscP2; DscP2	BB	Actinobacteria; Thermoleophilia; Gaiellales; uncultured; uncultured
14	BS	Aerophobetes; Aerophobetes; Aerophobetes; Aerophobetes; Aerophobetes;	BB	Planctomycetes; Phycisphaerae; MSBL9; SG8-4; SG8-4
15	BS		BB	Actinobacteria; Actinobacteria; Streptomycetales; Streptomycetaceae; *Streptomyces*
16			BB	Actinobacteria; Actinobacteria;

				Propionibacteriales; Propionibacteriaceae; *Microlunatus*
17			BB	Proteobacteria; Deltaproteobacteria; Desulfobacterales; Desulfobacteraceae; SEEP-SRB1
18			BB	Spirochaetes; Spirochaetia; Spirochaetales; Spirochaetaceae; uncultured

1.1 Supplementary Figures

Supplementary Figure 1. Principal coordinates analysis (PCoA) for archaea showing the similarity of samples from the two different sites and different depths. Similarity was determined by abundance-weighted metrics (Bray-Curtis). (A) PCoA plot. The loading explained by the two axes is shown (23% for x, 17% for y). Symbol size represents sample depth. (B) Secondary axis ordination (18% of variance) versus sample depth, with an exponential fit. Open symbols with solid outlines indicate Bering Sea samples. Gray symbols with dashed outlines indicate Bay of Bengal samples. (C) Same as (B) but the second PCoA axis (15% of variance).
