# Supplementary figures and images for: Microbial Selection and Survival in Subseafloor Sediment
Source: Front Microbiol. 2019 May 14;10:956. doi: 10.3389/fmicb.2019.00956 (PMC6527604; doi:10.3389/fmicb.2019.00956)

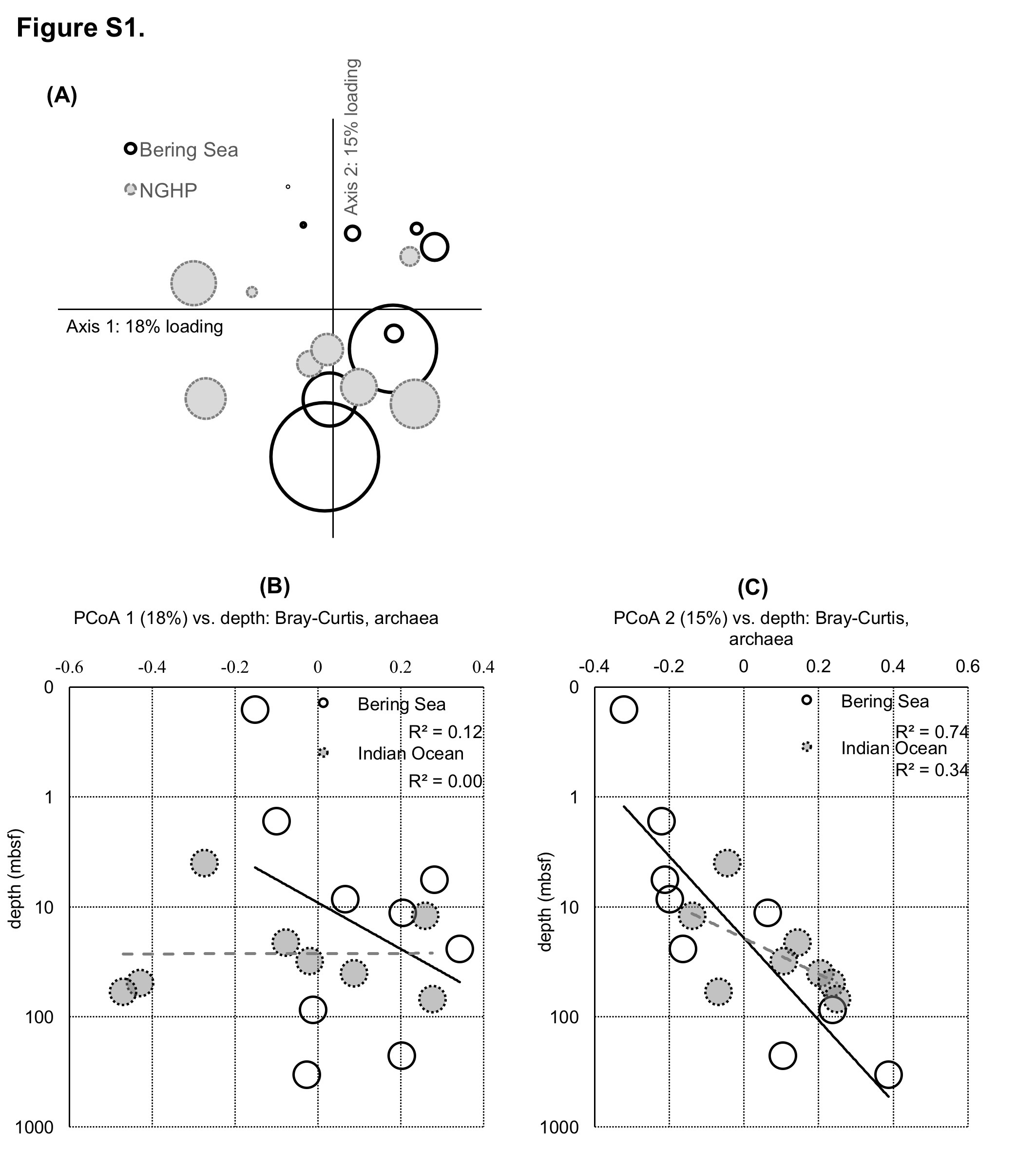

Supplement: Supplementary file 2 [file Image_1.JPEG]
